# Skeletal Manifestations of Scurvy: A Case Report from Dubai

**DOI:** 10.1155/2012/624628

**Published:** 2012-09-03

**Authors:** Shahryar Noordin, Naveed Baloch, Muhammad Sohail Salat, Abdul Rashid Memon, Tashfeen Ahmad

**Affiliations:** ^1^Department of Surgery, Section of Orthopaedics, Aga Khan University, P.O. Box 3500, Stadium Road, Karachi 74800, Pakistan; ^2^Department of Paediatrics and Child Health, Aga Khan University, Karachi 74800, Pakistan; ^3^Department of Biological and Biomedical Sciences, Section of Anatomy, Aga Khan University, Karachi 74800, Pakistan

## Abstract

*Introduction*. Nutritional deficiencies are rarely reported in developed countries. We report a child of Pakistani origin brought up in Dubai who developed skeletal manifestations of scurvy due to peculiar dietary habits. *Case Presentation*. A 4.5 year old boy presented with pain and swelling of multiple joints for three months and inability to walk for two months. Dietary history was significant for exclusive meat intake for the preceding two years. On examination the child's height and weight were below the 5th percentile for his age. He was pale and tachycardic. There was significant swelling and tenderness over the wrist, knee and ankle joints, along with painful restriction of motion. Basic blood workup was unremarkable except for anemia. However, X-rays showed delayed bone age, severe osteopenia of the long bones, epiphyseal separation, cortical thinning and dense zone of provisional calcification, suggesting a radiological diagnosis of scurvy. The child was started on vitamin C replacement therapy. Over the following two months, the pain and swelling substantially reduced and the child became able to walk. Repeat X-rays showed improvement in the bony abnormalities. *Conclusion*. Although scurvy is not a very commonly encountered entity in the modern era, inappropriate dietary intake can lead to skeletal abnormalities which may be confused with rickets. A high index of suspicion is thus required for prompt diagnosis of scurvy in patients with bone and joint symptoms.

## 1. Introduction

For the paediatric patient who presents with multiple bone and joint pains, the diagnosis usually rests among rickets, juvenile rheumatoid arthritis, and reactive arthritis [1]. However, the treating physician needs to elicit history of nutritional deficiencies especially in developing countries [2]. Even in developed countries, nutritional deficiencies can occur because children may have peculiar dietary habits [3]. We report a child of Pakistani origin settled in Dubai with severe bone and joint abnormalities resulting from a diet which was restricted to meat exclusively.

## 2. Case Presentation

### 2.1. History

A four-and-half-year-old male child was brought to the outpatient clinic with a history of pain and swelling of joints for three months and inability to walk and sit for two months.

Child was well three months before, when he had fever for a few days documented at 100-101°F, which responded to antipyretics. Subsequently his parents first noticed swelling at right wrist joint followed by left knee and left ankle joint without pain or restriction of joint motion. Gradually, pain started in these joints, and was mild in intensity, dull in nature, and aggravated by movement of limbs. After one month of onset of symptoms, the child developed difficulty in walking due to painful restriction of knee motion, such that he became bed- and chair-bound.

Past history was significant for lower limb weakness one year back which was diagnosed as Guillian Barre' Syndrome in another hospital. He was then treated with intravenous immunoglobulin for five days, and returned to normal health and activity within a month. Birth and vaccination history were insignificant. He was a student of class one.

Dietary history was significant in that the child was exclusively eating meat. There was no intake of vegetables, fruits, fresh juices, dairy products, or eggs. The family had moved from Pakistan to Dubai two years before, and the child's current dietary habits were established when they had settled in Dubai. The child had received treatment of various kinds in Dubai without improvement, and was then brought to the Aga Khan University Hospital in Karachi, Pakistan, for further management.

### 2.2. Examination

On general physical examination the child was sick looking, irritable, and pale.

His weight was below the 5th percentile, though height was above the 50th. Heart rate was 130 beats per minute, respiratory rate 32 breaths per minute, temperature 36.4°C, blood pressure 110/60 mmHg, and O_2_ saturation 98% on room air.

**Figure 1:**
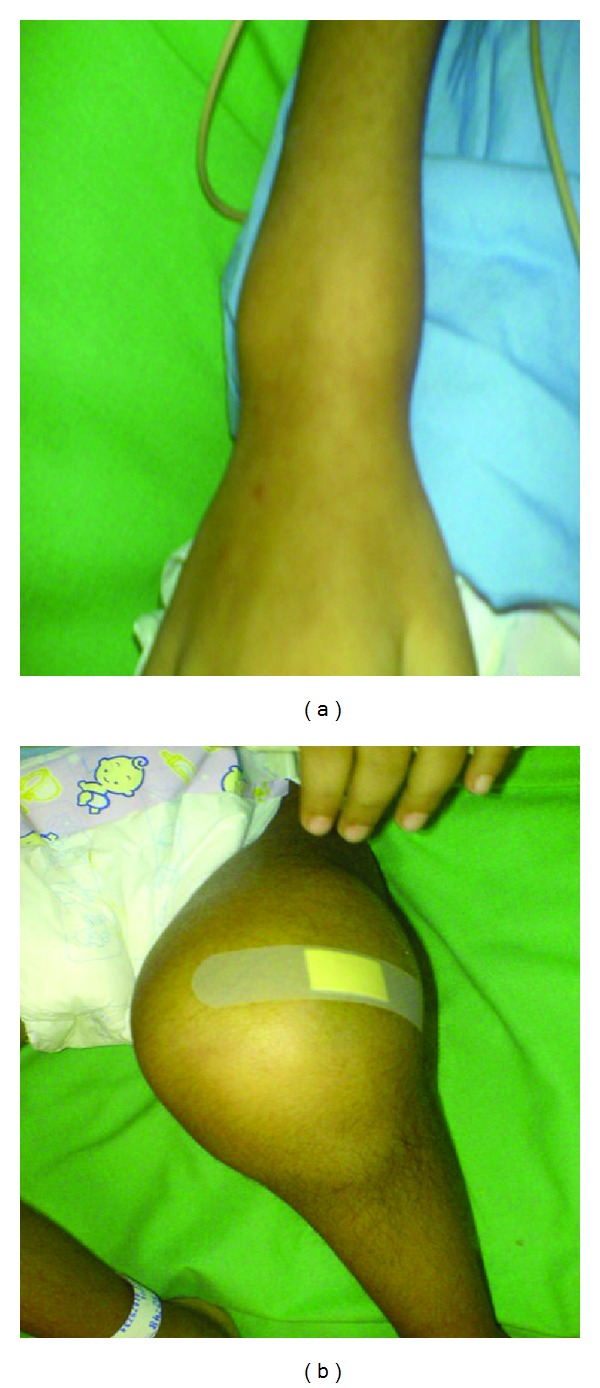
Clinical photographs showing swelling of the right wrist (a) and left knee (b).

On musculoskeletal examination, the child had swelling of wrist (Figure 1(a)) and knee joints (Figure 1(b)) bilaterally. The joints were tender and warm, though overlying skin was normal. Both knee joints had effusion and flexion contracture of 20° with further flexion possible only to 45° due to pain.

There were no abnormal findings on respiratory, cardiovascular, and abdominal examination.

### 2.3. Investigations

Laboratory studies performed at the time of admission revealed severe microcytic hypochromic anemia and vitamin D insufficiency (Table 1). Arthrocentesis was done from the left knee, and the culture report was negative, thus excluding septic arthritis.

**Figure 2:**
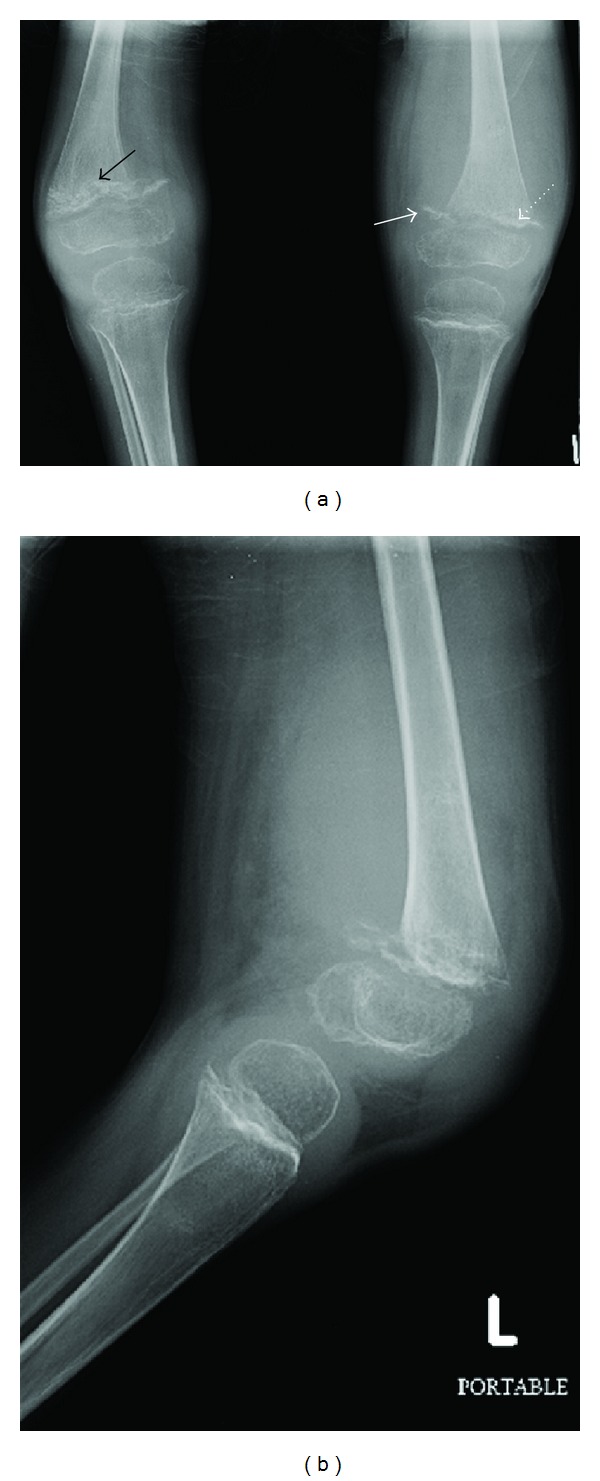
Anteroposterior (a) and lateral (b) X-rays of the knees showing varus deformity. Visible abnormalities include ground glass osteoporosis, thinning of the cortices, lucencies at metaphysis (Trummerfeld zone, black arrow), small corner fractures (Pelkan's spur, solid arrow), and dense zone of provisional calcification in the physis (White line of Frankel, dotted arrow).

X-rays of the knees (Figures 2(a) and 2(b)) showed ground glass osteoporosis, thinning of the cortices especially in the region of epiphysis (Ring sign), dense zone of provisional calcification at physis (White line of Frankel) with lucencies at metaphyses (Trummerfeld zone) of both femora and tibiae, and small corner fractures (Pelkan's spur).

**Figure 3:**
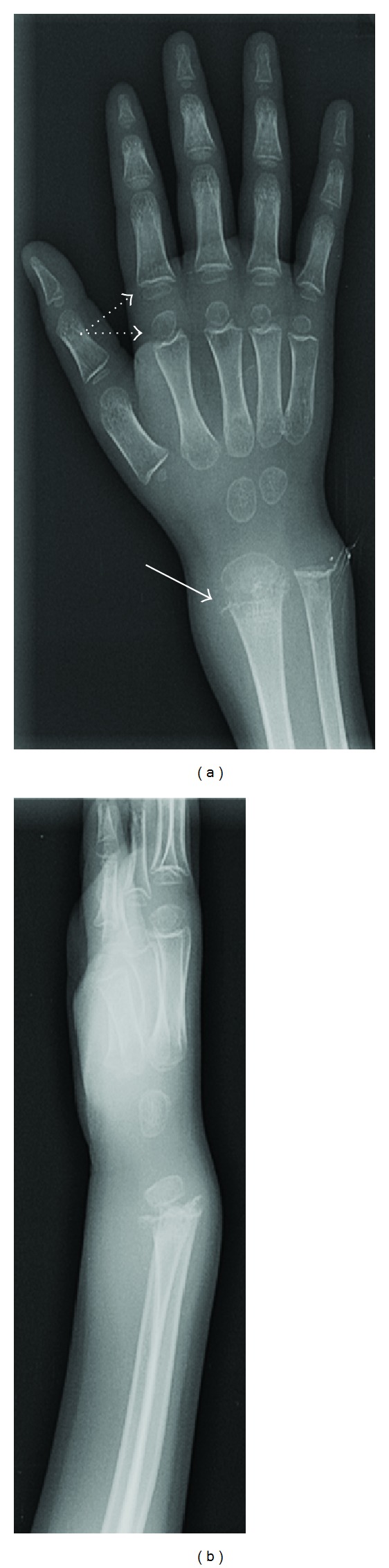
Anteroposterior (a) and lateral (b) X-rays of the hands and wrists showing severe osteopenia of the epiphyses of the phalanges (dotted arrow), metacarpals and the carpal bones, epiphyseal separation of the distal radius and ring sign (arrow).

X-rays of the wrist (Figures 3(a) and 3(b)) showed similar ground glass osteoporosis with metaphyseal abnormalities as in the knees, along with delayed bone age as only two carpal bones were visualized.

Findings were suggestive of Vitamin C deficiency (scurvy).

### 2.4. Management

The child was admitted in the hospital and after work up he was diagnosed to be suffering from scurvy. He was started on oral vitamin C replacement therapy in a dose of 250 mg daily. He was transfused packed red cells which brought hemoglobin level to 9.4 gm/dL. Oral supplementation of folic acid, iron, and multivitamins was added and the child was subsequently discharged.

**Figure 4:**
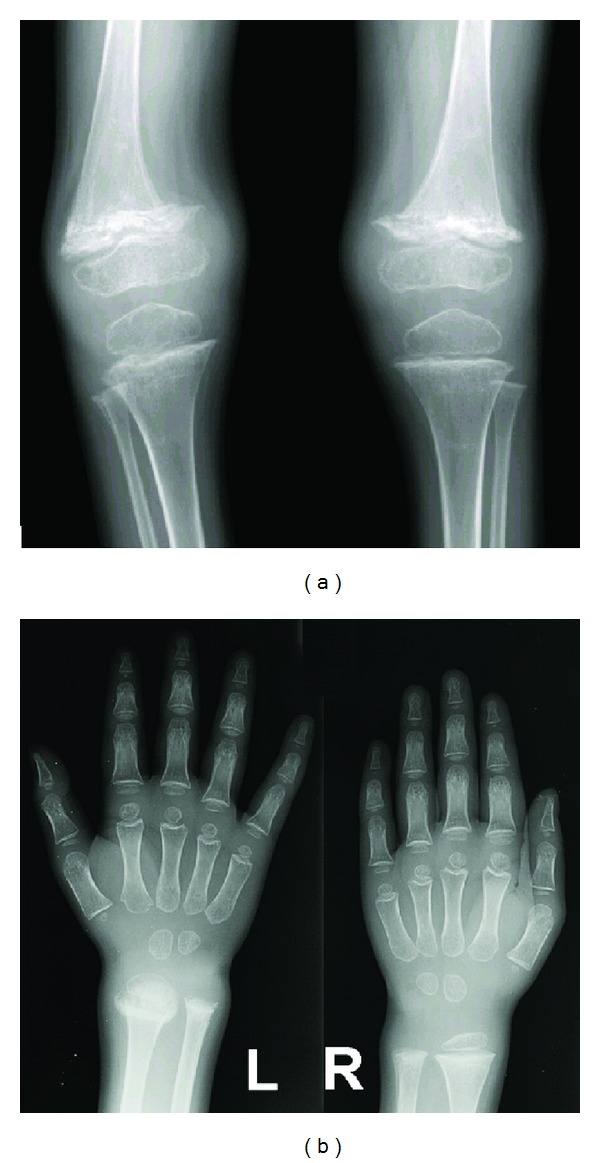
Follow-up X-rays of the knees (a) and wrist (b) at 6 weeks after initiation of vitamin C replacement therapy, showing improvement in osteopenia, healing of the epiphyseal fractures, and reduction in metaphyseal lucencies.

On follow-up visits he showed progressive improvement in the joint swellings and pain. The range of motion of the knee joints also improved, and he started walking after about four weeks of therapy. Follow-up X-rays (Figures 4(a) and 4(b)) performed 6 weeks after initiation of treatment confirmed adequate response to the treatment as evidenced by improvement in osteopenia, healing of the epiphyseal fractures, and reduction in metaphyseal lucencies.

The patient returned to Dubai after 10 weeks.

## 3. Discussion

In children, bone and joint pain and refusal to walk generally alerts the physician to possible osteomyelitis, septic arthritis, and rheumatic disorders [1]. When associated with bleeding manifestations, the bone pain may suggest acute leukemia. Scurvy is a rare cause of bone and joint symptoms, and this rarity of occurrence as compared to other nutritional deficiencies frequently leads to delayed recognition of this disorder [4, 5]. Initial manifestations of scurvy are vague and include irritability, decreased appetite, and delayed development [6]. As effects of vitamin C deficiency progress, affected children lie still with little movement because of generalized tenderness, most apparent in bones as a result of subperiosteal hemorrhages. Swelling may be noted along the shafts of long bones. Pseudoparalysis may be apparent as a result of the bone pain [6]. The dominant symptoms in our patient were joint swelling and pain. Through investigations, juvenile rheumatoid arthritis, and septic arthritis were excluded and finally a diagnosis of scurvy was made based on a combination of dietary history, clinical findings, and radiographic features.

Radiological changes in the long bones, particularly around the knee, are most diagnostic of scurvy. The bones of the child with scurvy demonstrate osteopenia [7] and easy fracturability (including Salter-Harris I fractures of the distal femur), often associated with vigorous callus formation [8]. The epiphyses and periosteum become easily detachable because of hemorrhage below the periosteum. Separation of the metaphyseal plate from the diaphysis, epiphyseal clefts, and malalignment of the metaphysis may also occur. In the X-rays of our patient also, the epiphyseal separation was apparent in the distal femur (Figure 2) and the radius (Figure 3). A circular, opaque radiologic shadow in the growth centers is often surrounded by a white line around the epiphysis, known as Wimberger ring sign [9], which is the normally mineralized cartilaginous zone of provisional calcification which contrasts more than normally with the adjacent demineralized osteopenic bone. This finding was also present in our patient, most notable in the distal radial epiphysis (Figure 3).

Cortical bone in vitamin C deficiency is characterized by thinning, which is sometimes described as a “pencil-point” cortex. Metaphyseal bone exhibits decreased trabeculae resulting in a decrease in radio-opacity similar to ground glass appearance, as seen in radiographs of our patient.

The physis exhibits thickening and sclerosis (Frankel line) which may be accompanied by a subjacent zone of lucency. This physeal thickening is known as a Frankel line, and the adjacent lucent zone on its diaphyseal side (secondary to poorly formed trabeculae) is known as the Trümmerfeld (German for “field of rubble”) zone or scurvy line [10], both these were present in radiographs of our patient (Figure 2). Its origin might be related to weakening of the metaphyseal region by osteoporosis that has undergone disruption by fracture and attempted repair.

The zone of proliferating cartilage cells is distorted, producing spicules from the metaphysis into the epiphyseal plate region. The zone of temporary calcification broadens, producing a wide, radiopaque metaphyseal band. Subjacent to this is a zone of poor-quality trabeculae, which appears radiolucent. A step-like lateral projection is found at the epiphyseal line in patients who are severely affected. Scorbutic skeletal changes are radiologically more severe in the lower extremities as evident in radiographs of our patient, whereas skeletal changes seen in rickets are allegedly more severe in the upper extremities.

Metaphyseal “beaks” and transverse lines of increased or decreased opacity may be seen in scurvy. The “beaks,” known as Pelkan spurs, are associated with healing fractures of the Trümmerfeld zone, at the periphery of the zone of calcification [11] as seen in initial and follow-up knee radiographs of our patient. They may be produced by lateral growth of the metaphyseal calcification zone and are associated with periosteal elevation.

Costochondral junctions of the first 6 or 8 thoracic ribs may be expanded; this change may be related to fracturing of the zone of provisional calcification during normal respiration. The costochondral junctions are rounded and appear smooth, knobby, and steplike. The enlargement of the costochondral junctions simulates that seen in rickets.

Skull changes may produce a “hair-on-end” or crew-cut appearance secondary to marrow hyperplasia in response to anemia. Examination of individuals with known scurvy reveals other skull changes (e.g., porotic hyperostosis, also described as a hair-on-end appearance, and marrow hyperplasia); however, no sphenoid changes are reported. Sphenoid porosity has not been shown to be caused by scurvy.

Subperiosteal hemorrhages are visualized only in the healing phase of scurvy, and these are almost invariably paraepiphyseal in distribution. Epiphyseal separation often results. Healing scurvy also appears with the loss of the scurvy line, in which the only residual manifestation is a double line of ossification at the original active site.

The diagnosis of scurvy is based on a combination of clinical and radiographic findings. A dietary history compatible with insufficient intake of vitamin C for at least 1–3 months should be present for signs and symptoms of scurvy to appear [12], and skeletal manifestations appear later. Dietary history of our patient also revealed exclusive intake of meat and lack of rich sources of vitamin C in the diet. Accurate laboratory measurement of vitamin C levels is difficult because of the instability of vitamin C, and Serum and plasma vitamin C measurements do not correlate well with tissue levels [13]. Anemia is known to be associated with scurvy, and was present in our patient [14], otherwise laboratory results were not helpful in arriving at a diagnosis.

Healing occurs rapidly with the oral administration of 100 to 200 mg/d of vitamin C. As healing occurs, the intake of vitamin C may be reduced to 50 mg/d until complete clinical and radiologic resolution has taken place [15]. Our patient showed rapid symptomatic improvement with replacement therapy, and was able to stand and walk in about four weeks after start of treatment.

## 4. Conclusion

Scurvy may occur in the modern era despite ready availability of fruits and vegetables, owing to peculiar dietary habits especially in the paediatric population. Physical signs of scurvy are often confused with rickets, a much more common condition in lower socioeconomic status regions. A high index of suspicion with a detailed dietary history is thus required for prompt diagnosis of scurvy in children with bone and joint symptoms in order to avoid unnecessary and expensive testing for rheumatological and connective tissue disorders.

## Figures and Tables

**Table 1:** Laboratory results.

Test	Result
Hemoglobin	5.4 (13.7–16.3 g/dL)
Total leucocyte count	15 (4–10 × 10^9^/L), 58% neutrophils (40–75%)
Platelet counts	535 (150–400 × 10^9^/L)
SGPT	8 (0–55 IU/L)
Alkaline phosphatase	144 (110–341 IU/L)
Calcium	9.3 (8.6–10.5 mg/dL)
Phosphorus	4.8 (2.7–4.8 mg/dL)
Uric acid	4.7 (<8.0 mg/dL)
Ferritin level	88 (7–140 ng/dL)
Vitamin D level	22 (30–100 ng/dL)
Antinuclear antibody	Negative
Rheumatoid factor	Negative
